# Clinical and Histopathological Variables and Prognostic Factors of Adrenocortical Carcinoma

**DOI:** 10.7759/cureus.15721

**Published:** 2021-06-17

**Authors:** Sara Sohail, Umal Azmat, Shehryar Khawaja, Khurram Mir, Muhammad Azam, Madiha Syed, Sajid Mushtaq, Usman Hassan, Ahmed Siddiqui, Syed Abbas Raza, Waqas Shafiq

**Affiliations:** 1 Endocrinology and Diabetes, Shaukat Khanum Memorial Cancer Hospital and Research Centre, Lahore, PAK; 2 Internal Medicine, Shaukat Khanum Memorial Cancer Hospital and Research Centre, Lahore, PAK; 3 Surgical Oncology, Shaukat Khanum Memorial Cancer Hospital and Research Centre, Lahore, PAK; 4 Pathology, Shaukat Khanum Memorial Cancer Hospital and Research Centre, Lahore, PAK

**Keywords:** adrenocortical carcinoma (acc), ki67 index, mitotane, mitotic rate, weiss score

## Abstract

Adrenocortical carcinoma (ACC) is a rare and highly aggressive tumor with a poor prognosis. The literature on prognosis from low-income or low-middle-income countries is limited and scarce. This study aimed to determine the clinical and histopathological characteristics, recurrence-free survival (RFS), overall survival (OS), and the factors affecting ACC's prognosis.

This was a retrospective study of patients that presented with ACC to the Shaukat Khanum Memorial Cancer & Research Center, Lahore, Pakistan, between January 2011 and May 2018. Information regarding demographics and clinical and histopathological variables were extracted and analyzed. Of the 25 subjects, 16 (64%) were female. The median age of the sample was 35 years (range; 21 - 72 years).

Statistically significant associations were found between RFS and functional status of the tumor (p = 0.014), cortisol overproduction (p = 0.02), androgen excess (testosterone [p = 0.03] and dehydroepiandrosterone sulfate [DHEA SO4] [p = 0.004]), Ki-67 score (p = 0.03), mitotic rate (p = 0.02), stratified mitotic rate (p = 0.01), and composite variable of disease (p = 0.004). The OS was found to have statistical associations with cortisol hypersecretion (p = 0.02), DHEA SO4 excess (p = 0.01), Modified Weis Score (p < 0.001), mitotic rate (p = 0.02), stratified mitotic rate (p = 0.003), and composite variable of disease (p = 0.001). Linear regression (forward-type) analysis suggested that the functional status of the tumor and the disease recurrence index statistically predicted the variance in RFS and OS, respectively.

Multiple clinical and histopathological variables appear to affect the prognosis of ACC. However, based on multivariable analysis, it appears that the functional status of the tumor and the composite variable of disease recurrence are predictors of RFS and OS, respectively.

## Introduction

Adrenocortical carcinoma (ACC) is a rare, highly aggressive malignancy with an incidence of 0.72 million persons per year [1]. It may present at any age; however, there is a bimodal age distribution. Incidence peaks are observed before the age of five years, and between 40-50 years of age [2]. Based on the functional nature of the tumor, ACC is categorized as secretory or non-secretory ACC. Within the secretory ACC sub-type, the majority of the tumors produce cortisol excess. The second most commonly produced hormones in patients with ACC are adrenal androgens, causing rapid-onset male pattern baldness, hirsutism, virilization, and menstrual irregularities in women. [3,4]. Radical open surgical excision of the primary tumor is the only curative therapy for ACC [5]. However, mitotane and radiation therapy have been used as adjuvant modalities [3,6].

The overall five-year survival of ACC is around 38-46 % [1,4]. There are only a handful of studies on the clinical and histopathological factors that may influence the prognosis of ACC worldwide [3,6,7]. However, the majority of these investigations have been conducted in the West. The literature from the low-income or low-middle-income countries is lacking, and in particular, in the South Asian population is scarce.

This study aimed to determine the clinical and histopathological characteristics, recurrence-free survival, overall survival, and to distinguish the factors affecting the prognosis of ACC.

## Materials and methods

A retrospective chart review of patients presenting with adrenocortical carcinoma (ACC) to the endocrinology clinic, Shaukat Khanum Memorial Cancer & Research Center, Lahore, Pakistan between January 2011 and May 2018, was carried out. The study was approved by the Institutional Review Board (IRB) of the Shaukat Khanum Memorial Cancer Hospital and Research Centre (EXMPT-17-07-18-01).

The patients underwent a comprehensive and thorough clinical assessment involving a detailed history, physical examination, and relevant laboratory and radiological investigations. The laboratory biochemical investigations consisted of complete blood count, urea and electrolytes, liver function test, 1 mg overnight dexamethasone suppression test, and aldosterone/renin ratio. In females, dehydroepiandrosterone sulfate (DHEA SO4) and testosterone levels were also assessed. Similarly, the histopathological assessment consisted of gross review, consisting of the size of the tumor, status of the capsule, presence of an area of necrosis, and extension of the tumor into adjacent adipose tissue. Likewise, the microscopic assessment included reviewing the percentage of clear cells, pure oncocytic population, and sarcomatoid areas, the number of mitosis per high-power field (HPF), atypical mitosis, areas of necrosis, capsular and vascular invasion, and this was used to determine the Modified Weiss scores. The Ki-67 stain was performed on all cases, and the Ki-67 index was determined on hotspots (the areas with maximum staining). The percentage of positive cells was calculated on microscopic images taken at 40x magnification. The patients did not undergo screening for genetic disorders.

All patients underwent cross-sectional imaging consisting of computed tomography (CT) or magnetic resonance imaging (MRI) of the chest, abdomen, and pelvis, or positron emission tomography (PET) scan.

The European Network for the Study of Adrenal Tumors (ENSAT) staging classification system was used to categorize the ACC. The ENSAT staging system defines stage I as ACC measuring less than or equal to 5 cm (in most significant dimension confined to the adrenal gland), stage II as tumor measuring greater than 5 cm without extra-adrenal invasion, and stage III as infiltration of disease into surrounding tissue, extension into the vasculature, or by the presence of disease in lymph nodes. Stage IV is described as a disease with distant metastasis [4].

All patients with stage I-III undergo radical surgical resection followed by surveillance. None of the patients receive mitotane therapy. The patients that experience recurrence of the disease, depending on the extent and nature of the recurrence, undergo surgery, chemotherapy (etoposide, doxorubicin, and cisplatin), or radiation therapy. Due to limited resources and expertise, patients with stage IV disease were not accepted in the hospital for treatment and hence not included in this study. 

All adult patients with histologically proven ACC were included in the investigation. Subjects less than 18 years of age were excluded. Data of the subjects was de-identified. The medical record was reviewed for demographics, weight alteration, presenting clinical characteristics such as abdominal pain, abdominal mass, hirsutism, menstrual irregularities, and cushingoid appearance (dorsocervical fat pads, round face, thinning of the skin, and easy bruising), and past medical history. The date of initial surgery, stage of the tumor at the time of diagnosis, pre-and post-surgery hormonal work-up, histopathological report, date of recurrence and subsequent treatments, last date of follow-up, and, if applicable, the date and cause of death were noted.

In this investigation, recurrence-free survival (RFS) was defined as the time between the initial surgery date and the date of relapse. Likewise, overall survival (OS) was described as the duration between the initial surgery date and the date of death or the last clinical follow-up. To assess the combined effect of tumor stage, mitotic rate, and Ki67, a predicting variable, termed as a composite variable of disease, was calculated. It was considered to be high risk if the subject had one or more of the following, ENSAT stage III, mitotic rate of 10/50 HPF or more, or Ki-67 score of 20 or more. 

The statistical analysis was performed using SPSS Statistics version 22.0 software (IBM Corp, Armonk, USA). A multistage analytic approach was practiced to assess the association between dependent and independent variables. In the first step, univariate associations were calculated using the Mann-Whitney U test and Chi-square test. If assumptions for the Chi-square test were not met, Fisher’s exact test was used. Similarly, where applicable, Pearson's two-tailed correlation coefficient was determined for continuous variables. If any observation was missing, it was excluded from the analysis. Additionally, a multiple linear regression analysis was conducted using a forward model approach to evaluate which independent clinical, biochemical, or histopathological characteristics could better explain the RFS and OS outcomes. A p-value of <.05 was considered statistically significant.

## Results

A total of 26 charts were identified and reviewed, and 25 subjects met the criteria for the study. One subject was excluded because of less than 18 years of age. Among the 25 subjects, 16 (64%) were female. The median age of the sample was 35 years (range, 21 -72 years). The most common presenting clinical feature was abdominal pain, which was reported by 17 (68%) subjects. Other common clinical features were hirsutism (56.3%), menstrual irregularities (50%), and hypertension (40%) (Table 1).

**Table 1:** Summary of study demographics and clinical characteristics

Study Characteristic	Category	Descriptive Statistic
Age (years)	Median	35 (21-72)
Gender	Male	9 (36%)
	Female	16 (64%)
Abdominal mass		6 (24%)
Abdominal pain		17 (68 %)
Hirsutism (females only) (n=16)		9 (56.3%)
Menstrual irregularities (n=16)		8 (50%)
Hypertension		10 (40%)
Diabetes mellitus		4 (18.2%)
Cushingoid appearance		2 (8%)
Hypokalemia		3 (12%)
Headaches		7 (28%)
Weight alteration		5 (20%)

Information regarding the biochemical and histopathological characteristics was also recorded. In 50% of the subjects, ACC was associated with hormonal overproduction. The breakdown of overproduction was as following: cortisol overproduction was found in nine subjects (81.8%), androgen excess was seen in five subjects (55.6%), and hypersecretion of mineralocorticoid was observed in two subjects (20%). All subjects underwent radical surgical resection of the primary tumor. At the time of presentation, the sample's median tumor size was 12.5 cm (range, 4 - 22 cm), and the most common ENSAT stage was II, which was seen in 68% (17) of the subjects. The median Modified Weiss score of the sample was 5 (range, 3 - 7). The median Ki-67 score of the cohort was 14 (range, 4.6 - 71.67), and in 30% (6) of the subjects, it was 20 or more. In 52.2% (12) of the subjects, the mitotic rate was 10 or more. Sixty percent (15) of the subjects were stratified as having a high risk for recurrence. However, 18 subjects had a recurrence of the disease. Among these, two subjects underwent repeat surgery, chemotherapy, and radiation therapy, three participants received chemotherapy alone, one subject underwent radiation therapy, and one had a repeat surgery alone. Nine cases did not receive active treatment and were referred to the palliative care service, and two subjects were lost to follow-up.

The median recurrence-free survival and the overall survival of the sample were 15 months (range, 2 - 77 months) and 24 months (2 - 92 months), respectively (Table 2).

**Table 2:** Summary of the functional status and the histopathological characteristics of the study population The functional status of the tumor was determined through documentation in the medical records. * None of the subjects had stage IV disease. ^ẟ^ It was considered to be high risk if the subject had one or more of the following: ENSAT stage III, mitotic rate of 10/50 HPF or more, or Ki-67 score of 20 or more. DHEA SO4: dehydroepiandrosterone sulfate; DST: dexamethasone suppression test; ENSAT: European Network for the Study of Adrenal Tumors; HPF: high-power field

Study Characteristic	Category	Descriptive Statistic
Functional Status	Secretory	12 (50%)
Testosterone	Elevated	5 (29.4%)
DHEA SO_4 _	Elevated	4 (25%)
1 mg overnight DST	Failed to suppress cortisol	8 (40%)
Aldosterone	Elevated	2 (9.5%)
Renin	Suppressed	2 (10%)
Modified Weiss score	Median	5 (3 - 7)
Tumor size (cm)	Median	12.5 (4 - 22)
Tumor stage *	Stage I	3 (12%)
	Stage II	17 (68%)
	Stage III	5 (20%)
Ki-67	Median	14 (4.6 - 71.67)
Stratified Ki-67	≥ 20	6 (30%)
Mitotic rate	Median	10 (4 - 33)
Stratified mitotic rate	≥ 10	12 (52.2%)
Composite variable of disease	High risk^ ẟ^	15 (60%)
Recurrence-free survival (months)	Median	15 (2 - 77)
Overall survival (months)	Median	24 (2 - 92)

The breakdown of statistical associations between clinical characteristics of the study population and RFS and OS are given in Table 3. The cushingoid appearance was found to be statistically associated with both RFS (p = 0.04) and OS (p ≤ 0.01).

**Table 3:** Breakdown of associations between recurrence free survival (RFS) and overall survival (OS) and study demographic and clinic characteristics * Correlation coefficient

Study Characteristic		RFS		OS	
		Median (Range) / Correlation Coefficient	P-Value	Median (Range) / Correlation Coefficient	P-Value
Age (years) *		.068	.64	.027	.85
Gender	Male	18 (4 - 77)	.12	24 (5 - 92)	.33
	Female	14 (2 - 48)		22 (2 - 56)	
Abdominal mass	Yes	12.5 (3 - 77)	.88	21 (7 - 77)	.88
	No	15 (2 - 76)		25 (2 - 92)	
Abdominal pain	Yes	15 (2 - 77)	.89	25 (4 -92)	.41
	No	15 (2 - 76)		20 (2 - 77)	
Hirsutism (females only)	Yes	6 (2 - 39)	.35	25 (2 -56)	.84
	No	14 (4 - 48)		19 (4 - 53)	
Menstrual irregularities (females only)	Yes	9.5 (2 - 39)	.51	14 (4 - 39)	.13
	No	11.5 (2 - 48)		32 (2 - 56)	
Hypertension	Yes	11.5 (2 - 77)	.81	24.5 (2 -77)	.77
	No	16 (2 - 54)		18 (4 - 92)	
Diabetes mellitus	Yes	4 (2 - 15)	.05	10.5 (2 - 25)	.10
	No	17 (3 - 76)		30 (4 - 77)	
Cushingoid appearance	Yes	3 (2 - 4)	.04	3 (2 - 4)	< .01
	No	16 (2 - 77)		25 (4 - 92)	
Hypokalemia	Yes	8 (2 - 48)	.72	19 (2 - 53)	.72
	No	15.5 (2 - 77)		24.5 (4 - 92)	
Headaches	Yes	14 (2 - 48)	.72	24 (15 - 53)	.66
	No	15.5 (2 - 77)		21.5 (2 - 92)	
Weight alteration	Yes	15 (3 - 77)	.55	24 (5 - 77)	.41
	No	17 (2 - 54)		21.5 (2 - 92)	

Statistically significant associations were found between RFS and functional status of the tumor (p = 0.014), cortisol overproduction (p = 0.02), androgen excess (testosterone (p = 0.03) and DHEA SO4 (p = 0.004), Ki-67 score (p = 0.03), mitotic rate (p = 0.02), stratified mitotic rate (p = 0.01), and composite variable of disease (p = 0.004). Similarly, OS was found to have statistical associations with cortisol hypersecretion (p = 0.02), DHEA SO4 excess (p = 0.01), Modified Weiss score (p < 0.001), mitotic rate (p = 0.02), stratified mitotic rate (p = 0.003) and composite variable of disease (p = 0.001). These results are summarized in Table 4. 

**Table 4:** Breakdown of associations between recurrence free survival (RFS) and overall survival (OS) and functional status and histopathological characteristics The functional status of the tumor was determined through documentation in the medical records. * Correlation coefficient ^ẟ^ It was considered to be high risk if the subject had one or more of the following: ENSAT stage III, mitotic rate of 10/50 HPF or more, or Ki-67 score of 20 or more. DHEA SO4: dehydroepiandrosterone sulphate; DST: dexamethasone suppression test; ENSAT: European Network for the Study of Adrenal Tumors; HPF: high-power field

Study Characteristic		RFS		OS	
		Median (Range) / Correlation Coefficient	P-Value	Median (Range) / Correlation Coefficient	P-value
Functional Status	Secretory	5.5 (2 - 48)	.014	15 (2 - 56)	.101
	Non-secretory	35.5 (6 - 54)		38.5 (11 - 92)	
Testosterone (females only)	Normal	29 (4 - 39)	.03	36 (4 - 44)	.34
	Elevated	3 (2 - 6)		13 (2 - 56)	
DHEA SO_4_	Normal	31.5 (4 - 76)	.004	38.5 (4 -92)	.01
	Raised	2.5 (2 - 6)		8.5 (2 - 17)	
Cortisol secretion	Normal	31.5 (6 - 48)	.02	38.5 (11 - 56)	.02
	Hypersecretion	4.5 (2 - 76)		10 (4 - 77)	
Aldosterone	Normal	11 (2 - 54)	.40	24.5 (2 - 92)	.47
	Elevated	28 (8 - 48)		36 (19 - 53)	
Renin	Normal	11 (2 - 54)	.38	24.5 (2 - 92)	.52
	Suppressed	28 (8 - 48)		36 (19 - 53)	
Modified Weiss score *		.101	.55	.763	< .001
Tumor Size *		.05	.75	.18	.24
Tumor Stage	Stage I	16 (6 - 39)	.40	16 (11 - 39)	.32
	Stage II	18 (2 - 77)		36 (4 - 92)	
	Stage III	7 (2 - 37)		19 (2 - 38)	
Ki-67 *		-.36	.03	-.27	.10
Stratified Ki-67	< 20	23.5 (2 - 77)	.08	37 (2 - 77)	.15
	≥ 20	5.5 (2 - 54)		15 (4 - 92)	
Mitotic rate *		-.371	.02	-.36	.02
Stratified mitotic rate	< 10	39 (5 - 77)	.01	44 (7 - 92)	.003
	≥ 10	7.5 (2 - 37)		16.5 (4 - 38)	
Composite variable of disease	Low risk	39 (6 - 77)	.004	46 (11 - 77)	.001
	High risk ^ẟ^	7 (2 - 54)		16 (2 - 92)	

A forward model multiple linear regression analysis indicated a model to account for the statistically significant portions of the variance of RFS and OS. The model for RFS suggested that the functional status of the tumor was the principal variable. The variance of this model was 82% (Table 5). On the contrary, the OS model suggested that the composite variable of disease was the distinctive variable. The variance of this model was 66% (Table 6).

**Table 5:** Multiple linear regression model assessing demographic, clinical, and histopathological characteristics for recurrence free survival (RFS) * Dependent variable was RFS; R2 was 0.82 and adjusted R2 was 0.798.

Characteristic	Standard B coefficient	Significance	Confidence interval (95%)	
			Lower	Upper
Functional Status of the tumor	.906	.000	16.32	36.48

**Table 6:** Multiple linear regression model assessing demographic, clinical, and histopathological characteristics for overall survival (OS) * Dependent variable was OS; R2 was 0.66 and adjusted R2 was 0.632.

Characteristic	Standard B coefficient	Significance	Confidence interval (95%)	
			Lower	Upper
Composite Variable of Disease	-.813	.000	- 49.7	- 18.8

## Discussion

This study's purpose was to determine the clinical and histopathological characteristics, recurrence-free and overall survival, and distinguish the factors affecting ACC's prognosis. The median RFS of the sample was 15 months (range, 2 - 77 months), and the median OS of the subjects was 24 months (range, 2 - 92 months). The RFS was found to be statistically associated with clinical cushingoid appearance (p = 0.04), hormone overproduction (p = 0.014), cortisol excess (p = 0.02), androgen hypersecretion (p ≤ 0.05), Ki-67 (p = 0.03), mitotic rate (p = 0.02), stratified mitotic rate (p = 0.01), and composite variable of disease (p ≤ 0.01). Similarly, the OS was significantly associated with clinical cushingoid appearance (p ≤ 0.01), cortisol excess (p = 0.02), DHEA SO4 overproduction (p = 0.01), Modified Weiss score (p ≤ 0.01), mitotic rate (p = 0.02), stratified mitotic rate (p ≤ 0.01), composite variable of disease (p ≤ 0.01). However, when analyzed using a linear regression (forward type) analysis, only the functional status of the tumor (secretory), and the composite variable of disease (high risk), were found to be statistically significantly associated with the RFS and the OS, respectively. This model approach suggested that the functional status of the tumor could account for 82% of the variability in RFS, and the recurrence risk factor could account for 66% of the variability in OS.

The median age of the cohort at the time of the presentation, in the present study, was 35 years (range, 21 - 72 years). On the contrary, other investigators have reported a median age at the time of presentation between 42 - 48.5 years [3,6-9]. The exact reason for an earlier presentation is unknown and beyond the scope of this study. However, it could be associated with the size of the tumor. The median size of tumors among subjects in the present study was 12.5 cm (range, 4 - 22 cm). On the other hand, the median size of tumors, amongst other investigations, was relatively less than 10 - 11.8 cm [2,3,9].

The most common reasons for presentation to the hospital among the study sample were abdominal pain, hirsutism, and menstrual irregularities. These findings are similar to those reported by the other investigators [3]. Abdominal pain in ACC is likely to be associated with the tumor's mass effect or secondarily from tissue damage associated with the localized invasion of the cancer cells into the visceral tissue. Similarly, hormonal over secretion is probably associated with hirsutism and menstrual irregularities amongst females.

The median RFS of the study sample was 15 months (range, 2 - 77 months). Previously, other investigators have reported median RFS between 10 - 42 months [3,6-9]. A notable reason for this disparity in reporting is likely to be associated with the therapeutic use of mitotane in patients with radically resected ACC. Participants undergoing mitotane therapy had a much longer RFS than those who did not receive this therapy [9]. In the present study, none of the subjects received mitotane therapy. Another possible reason for the difference in RFS could be associated with the stage of ACC. Studies in which most of the sample consisted of earlier-stage disease had relatively higher RFS compared to those with a larger composition of subjects with late-stage disease [3].

The median OS of the cohort was 24 months (range, 2 - 92 months). On the contrary, other investigators have reported a much longer median OS, ranging from 35.2 - 110 months [3,6-8]. An important reason for this meaningful difference is likely to be associated with the use of mitotane therapy. Unlike in the present study, prior investigators have used mitotane therapy either as adjuvant therapy or as part of multimodal therapy in subjects with recurrence [9].

The linear regression (forward type) analysis model suggested that the functional status of the tumor (secretory) was statistically associated with poor RFS. The variance of this model was 82%. In the present study, two-thirds of the participants with hypersecretory tumors had cortisol excess. Prior studies have reported that cortisol hypersecretion is associated with poor prognosis among patients with ACC [3,10]. The exact reason for this affiliation is unknown. However, multiple hypotheses have been suggested, such as comorbidity due to Cushing's syndrome, immunosuppressive effects of cortisol excess, which could promote tumor progression and metastasis, and the underlying pathophysiology of the hypersecreting cortisol tumor [10-14].

The linear regression model (forward type) determined that the OS of ACC was significantly associated with the composite variable of disease. This model accounted for 66% of the variability in OS. The composite variable of disease was a prediction index consisting of Ki-67 index, mitotic count, and tumor stage. It was considered to be high risk if a participant had either a Ki-67 score of ≥20, or mitotic rate of 10/50 HPF or more, or ENSAT stage III. Individually, multiple studies have suggested that these variables are inversely proportional to OS [3,4,6-8,12,15]. Both the Ki-67 index and mitotic rate are markers of the aggressiveness of the tumor. Likewise, the stage reflects the extent of the spread.

A limitation of the study was the inherent study design. Retrospective studies are considered low-quality in the hierarchy of evidence due to lack of blindness. They have a high potential for recall bias, reconfirmation bias, and selection bias. Overall this may result in false-positive associations. Nonetheless, all of the information was extracted from the computerized hospital database, and data from other medical providers were correlated to reduce the risk of recall and reporting bias. Another limitation is that this was a single-center study, which may affect the generalizability of the findings. However, this study was conducted in a tertiary care center, where patients were referred for treatment from across the region, including Afghanistan. Nevertheless, these results need to be interpreted with caution and necessitate replication. Future investigations with prospective study design, multi-center enrollment, larger sample sizes, which use adjuvant treatments in high-risk groups, are advocated.

## Conclusions

The prognosis of ACC remains poor. Multiple clinical and histopathological variables appear to affect the outcome of ACC. However, based on multivariable analysis, it appears that the functional status of the tumor and the composite variable of disease recurrence are predictors of recurrence-free and overall survival, respectively.
